# TRPV4 Regulates Imiquimod (IMQ)-Induced Psoriasis via CaMKKβ/AMPK and Calmodulin/Akt-Mediated Autophagic Signaling Pathways in Juvenile Murine Keratinocytes

**DOI:** 10.3390/dermatopathology13030041

**Published:** 2026-09-05

**Authors:** Qing Xie, Di Bao, Tao Ruan, Kuangzheng Zhu, Dawei Liu, Tiecheng Zhong

**Affiliations:** 1 Ascletis BioScience Co., Ltd., Hangzhou 310018, China; 2 Chia Tai Tianqing Pharmaceutical Group Co., Ltd., Nanjing 211100, China; 3 Hulk Cloud-Wise (Hangzhou) Technology Co., Ltd., Hangzhou 310052, China; taoruan@outlook.com; 4 Hangzhou Charm Pharmaceuticals, Ltd., Headquarters Economy Park for Alumni Corporation of Zhejiang University, Hangzhou 311000, China; 5 Hangzhou Chasing Pharmaceutical Co., Ltd., Hangzhou 311400, China

**Keywords:** psoriasis, transient receptor potential vanilloid 4 (TRPV4), autophagy, reactive oxygen species (ROS), CaMKKβ/AMPK signaling pathway, Calmodulin/Akt signaling pathway

## Abstract

**Background:** Psoriasis is a chronic inflammatory skin disease due to genetic susceptibility and multiple environmental factors. Transient receptor potential vanilloid 4 (TRPV4) is a calcium channel extensively expressed throughout the body with multiple functions. However, the role of TRPV4 in imiquimod (IMQ)-induced -psoriasis progression remains unclear although it is ubiquitously expressed in skin tissues and participates in cutaneous mechano- and thermo-sensation. **Methods:** Both in vivo and in vitro studies were performed in murine skin tissue and isolated cells. TRPV4 expression, autophagy, and Akt/mTOR phosphorylation were assessed by Western blot and reactive oxygen species (ROS) generation was examined by MitoSOX staining. Cytokine levels were measured by ELISA and cell proliferation was determined by CCK staining. The severity of psoriasis was also checked by psoriasis severity index (PSI), scale and thickness scoring and microscopic observations. **Results:** IMQ-induced psoriasis was remarkably attenuated in TRPV4 knockout mice compared with their wild-type counterparts, accompanied by reduced expression of several inflammatory cytokines. Deletion of TRPV4 diminished ROS production and Akt/mTOR phosphorylation. In vitro experiments showed that IMQ caused a concentration-dependent increase in autophagy, characterized by an initial increasing phase followed by a declining phase in primary keratinocytes. IMQ-induced autophagic activity was enhanced via Calmodulin/Akt pathway suppression while weakened by CaMKKβ/AMPK pathway inhibition. Moreover, blockade of the Calmodulin/Akt pathway significantly reduced IMQ-induced ROS production and psoriasis severity, whereas inhibition of the CaMKKβ/AMPK pathway exacerbated IMQ-induced psoriasis progression through ROS accumulation. **Conclusions:** Both CaMKKβ/AMPK and Calmodulin/Akt signaling pathways, as downstream components of TRPV4, are involved in IMQ-induced psoriasis: lower IMQ dosage activates AMPK, thereby preventing psoriasis by ROS clearance, whereas higher IMQ dosage activates Calmodulin and exacerbates psoriasis. Our findings reveal the potential values of targeting TRP-related channels in IMQ-induced psoriasis.

## Simple Summary

The present study supports the model that Calmodulin/Akt and CaMKKβ/AMPK pathways exert distinct and opposing effects during IMQ-induced psoriasis through regulation of autophagy-dependent mitochondrial ROS clearance, representing important downstream mediators of TRPV4 signaling pathways and may provide promising therapeutic approaches for psoriasis treatment in rodent animals.

## 1. Introduction

Psoriasis is a chronic inflammatory skin disorder caused by combined effects of multiple environmental factors and activities of various cell types. It is also one of the most common refractory dermatological diseases, with a global epidemiology prevalence ranging from approximately 2% to 3% [1]. The disease is characterized by abnormal proliferation of keratinocytes (KCs), infiltration of inflammatory cells, hyperplasia and dermal papillary blood vessels dilation, leading to a series of pathophysiological alterations, including hyperkeratosis or parakeratosis, loss of the granular layer, and acanthosis. Immunocyte-mediated immunity plays a pivotal role in both the initiation and maintenance of psoriasis [2]. Consequently, the prevention and treatment of psoriasis have become a recognized global challenge, attracting increasing attention from both the scientific and clinical communities.

Reactive oxygen species (ROS) play an indispensable role in the pathogenesis of psoriasis through several mechanisms. (1) Excessive ROS induce oxidative stress and disrupt skin homeostasis. Elevated ROS levels damage lipids, proteins, and DNA, thereby impairing epidermal barriers and creating favorable conditions for persistent inflammation. (2) ROS promote abnormal KCs proliferation and differentiation disorders through activation of a series of signaling pathways, including mitogen-activated protein kinase (MAPK) and nuclear factor-kappa B (NF-κB). ROS also stimulate excessive proliferation while inhibiting normal differentiation of KCs, thereby exacerbating epidermal hyperplasia and hyperkeratosis. (3) ROS amplify inflammatory responses and establish vicious cycles by inducing the release of numerous cytokines and chemokines in KCs, such as TNF-α, IL-6, IL-8, IL-17, and IL-23, promoting the recruitment and activation of inflammatory cells like T cells, neutrophils, etc. KCs are critically involved in the development and progression of the disease through the secretion of pro-inflammatory mediators such as IL-1β, tumor necrosis factor-alpha TNF-α, and IL-36, which activate plasmacytoid and myeloid dendritic cells (DCs). Activated DCs produce high levels of IL-23, a cytokine essential for the survival and maintenance of Th17 lymphocytes [3]. (4) ROS contribute to immune dysregulation and T-cell polarization. (5) ROS participate in angiogenesis and dermal remodeling. Inflammatory cells recruited to psoriatic lesions generate additional ROS, forming a positive feedback loop between inflammation and oxidative stress, further facilitating Th1/Th17 responses while suppressing regulatory T-cell (T-reg) function, thereby driving psoriasis-associated immune imbalance [4,5]. To counteract these detrimental effects, autophagy serves as an essential cellular protective mechanism by removing accumulated waste products and harmful substances, including damaged proteins, organelles, and mitochondria that are major sources of ROS production. Mounting evidence has demonstrated crucial roles of autophagy in the pathophysiological progression of psoriasis. However, the intracellular mechanisms by which damaged mitochondria accumulate and modulate ROS production through autophagic alterations within skin tissues still remain incompletely understood.

Accumulating evidence indicates that transient receptor potential (TRP) channels are associated with a variety of skin disorders, including atopic dermatitis, psoriasis, and cutaneous malignancies [6,7]. For example, attenuation of psoriasiform dermatitis in TRPV1 knockout (KO) mice was observed and associated with reduced angiogenesis, decreased inflammatory infiltration, and lower levels of psoriasis-related cytokines [6]. Among those TRP family channels, transient receptor potential vanilloid 4 (TRPV4), a member of the TRPV subfamily which is abundantly expressed in skin tissues, plays important regulatory roles and participates in a variety of physiological functions [8,9]. TRPV4 is a non-selective cation channel with moderate calcium selectivity [10]. Under certain physiological and pathological conditions, TRPV4 expression is markedly upregulated, and its downstream signaling pathways include, but are not limited to: AMP-activated protein kinase (AMPK), protease-activated receptor 2 (PAR2), phosphoinositide 3-kinase (PI_3_K), calpain, extracellular signal-regulated kinase 1/2 (ERK1/2), nuclear factor of activated T cells cytoplasmic 1 (NFATC1)/myocyte enhancer factor 2C (MEF2C), and adenosine triphosphate (ATP). Those signaling molecules influence cellular differentiation, proliferation, apoptosis, autophagy, protein synthesis, and migration [11]. TRPV4 is identified as a key regulator of skin barrier homeostasis, cellular proliferation, and pruritic responses since it could get activated by certain physical and chemical stimuli along with inflammatory metabolites, [8,9,12,13]. In murine models, it leads to the overexpression of ATP derived from KCs, and initiates enlarged IL-23/Th17 responses in IMQ-induced psoriasis [14]. While TRPV4 activation is known to stimulate a pleiotropic range of above-mentioned downstream signaling cascades, the precise mechanistic axes driving psoriasis remain incompletely understood.

Since the primary function of TRPV4 is to mediate Ca^2+^ influx, we postulate that the most critical downstream effectors responsible for psoriasis progression would be directly governed by intracellular calcium sensors. Calmodulin is a ubiquitous Ca^2+^-binding protein that acts as a primary transductor of intracellular calcium signals and it directly binds to and activates specific downstream kinases upon Ca^2+^ stimulation, most notably Calmodulin-dependent CaMKKβ and Akt. CaMKKβ is a well-established upstream activator of AMPK, a critical regulator of metabolic stress. Concurrently, the Calmodulin-dependent activation of Akt regulates cell proliferation [15,16]. Therefore, we narrowed the scope into CaMKKβ/AMPK and Calmodulin/Akt signaling pathways as downstream components of TRPV4 for further investigation. Therefore, the primary objective of the present study was to verify the hypothesis that modulation of the TRPV4-associated signaling pathway participates in the progression of imiquimod (IMQ)-induced psoriasis through regulation of autophagy-mediated mitochondrial ROS production. Based on the current evidence, we proposed the following hypotheses: (1) TRPV4 activation contributes to IMQ-induced psoriasis by regulating mitochondrial ROS production via autophagic activity; (2) activations of the CaMKKβ/AMPK and Calmodulin/Akt downstream signaling pathways affect the initiation and progression of IMQ-induced psoriasis; and (3) genetic modifications targeting TRPV4 expression might represent a novel therapeutic strategy for psoriasis prevention and treatment in animals.

## 2. Materials and Methods

### 2.1. Reagents and Chemicals

MitoSOX™ Red as a mitochondrial superoxide indicator (M-36008) was purchased from Thermo Fisher Scientific (Waltham, MA, USA). Primary antibodies against β-actin (#4967) and LC3 (#2775) were obtained from Cell Signaling Technology (Danvers, MA, USA). Antibodies against mTOR (PA1-518), phosphorylated mTOR (p-mTOR; 44-1125G), Akt (MA5-14916), phosphorylated Akt (p-Akt; MA5-51537), and TRPV4 (PA5-41066) were purchased from Thermo Fisher Scientific (Waltham, MA, USA). ELISA kits for TNF-α (ml002095), IL-17 (IL-17F, ml063129), and IL-23 (ml063140) were obtained from Shanghai Enzyme-linked Biotechnology Co., Ltd. (Shanghai, China), while the IFN-γ ELISA kit (98023ES48) was purchased from Yeasen Biotechnology Co., Ltd. (Shanghai, China). Imiquimod cream (IMQ; approval No. H20030129) was supplied by Sichuan Med-Shine Pharmaceutical Co., Ltd. (Chengdu, China). AZD5363 (HY-15431), IMQ powder (HY-B0180), Compound C (CC; HY-13418A), STO-609 (HY-19805), and W-7 (HY-130368) were purchased from MedChem Express (Monmouth Junction, NJ, USA).

### 2.2. Experimental Animals

All animal experiments were performed using 6~8-week-old juvenile C57BL/6NCya mice purchased from Cyagen Biosciences Inc. (Santa Clara, CA, USA). TRPV4 knockout (KO) mice were generated as previously described (Suzhou, China; S-KO-11372; production license No. SCXK 2025-0021). Age-matched wild-type (WT) C57BL/6 mice also purchased from Cyagen Biosciences Inc. were used as negative controls [17,18]. Mice were housed at the Animal Laboratory Center of Zhejiang University under specific pathogen-free (SPF) conditions. All experimental procedures were approved by the IACUC of Zhejiang University (Approval No. 15110) and conducted in accordance with the institutional guidelines.

### 2.3. Cell Culture

Primary KCs were isolated from the skin of WT mice used in Section 2.2. Briefly, skin tissues were excised, and subcutaneous tissues were carefully removed before enzymatic digestion. Epidermal KCs were isolated, filtered, and collected by centrifugation. Cells were resuspended and cultured in a low-calcium medium containing 8% fetal bovine serum (FBS) and 0.05 mM Ca^2+^. Cultures were maintained at 37 °C in a humidified incubator with 5% CO_2_. The culture medium was replaced every 48–72 h. When cells reached 80~90% confluence, they were passaged at a ratio of 1:4–1:5 and used within eight passages. To harvest qualified KCs with desired purity, it should be noticed that the majority of non-KCs initially attached to the plate already differentiated and switched to detached states following a 4 day culture, leaving the remaining attached KCs with a typical cobblestone morphology, and those KCs could proliferate and not respond to suprabasal differentiation markers. There might be noticeable signs of senescence such as morphological heterogeneity on the 10th day. Thus, it is preferred to utilize the cells between Day 4 and Day 7 to ensure the reproductivity [19].

### 2.4. Establishment of the Psoriasis Model and Disease Scoring in Mice

Psoriasiform dermatitis was induced using IMQ as previously described [2]. Briefly, each cohort of male WT and TRPV KO mice were randomly divided into two groups by weight (6 mice in each group) with one group receiving IMQ while the other group receives vehicle (plain cream without IMQ). The dorsal hair of mice was shaved with a trimmer to expose the skin and 62.5 mg of 5% IMQ cream was applied daily to a 5 cm^2^ shaved area on the dorsal skin of each mouse for 7 consecutive days (daily dose 3.125 mg). The severity of skin inflammation was assessed using the Psoriasis Severity Index (PSI) score evaluation, including erythema, scaling, and epidermal thickness (in μm), according to previously established criteria [14,20]. Individual scores were recorded and analyzed quantitatively.

### 2.5. Western Blot Analysis

Protein expression levels were determined by Western blot. Primary antibodies against TRPV4, phosphorylated Akt (p-Akt), total Akt, phosphorylated mTOR (p-mTOR), total mTOR, and LC3 were used at a dilution of 1:1000. β-actin served as the internal loading control. Horseradish peroxidase (HRP)-conjugated anti-rabbit IgG and anti-mouse IgG secondary antibodies were applied at a dilution of 1:12,000. Protein bands were visualized via enhanced chemiluminescence and quantified using ImageJ software (Version 1.49v, National Institutes of Health, Bethesda, MD, USA).

### 2.6. Measurement of In Vivo Psoriasis-Related Cytokines by ELISA

The concentrations of TNF-α, IL-17, IL-23, and IFN-γ in murine serum were determined using commercially available ELISA kits according to the manufacturers’ instructions. Briefly, let the blood sample clot naturally at room temperature for 10–20 min, then centrifuge the sample for approximately 20 min (2000–3000 rpm) at 4 °C and collect the supernatant for further analysis. Cytokine levels in the resulting supernatant were measured by enzyme-linked immunosorbent assay (ELISA) following the manufacturer’s instructions. The samples were incubated with the indicated antibody-coated plates and horseradish peroxidase (HRP)-labeled secondary antibody at 37 °C for 1 h. After incubation with chromogenic agents A and B at 37 °C for 15 min, the OD values were measured by a microplate reader at 450 nm, and standard curves were generated by linear regression analysis using the reference samples inside the kit with different concentrations. Cytokine concentrations in the samples were calculated based on the corresponding standard curves.

### 2.7. Cell Counting Kit-8 (CCK-8) Assay

Primary KCs were seeded into 96-well plates with a density of 1 × 10^4^ cells/mL (100 μL per well). After 24 h of incubation to allow cell attachment, the culture medium was replaced with serum-free medium for an additional 24 h. Cells were subsequently treated with different concentrations of test compounds, with each condition performed in triplicate, and incubated for another 24 h. Thereafter, 10 μL of CCK-8 reagent was added into each well and incubated in the dark for 1.5 h. Absorbance was measured at 450 nm using a microplate reader. Cell viability and proliferative activity were calculated based on the generated standard curves.

### 2.8. Detection of ROS Levels in Murine Skin Tissues and Primary KCs

For tissue ROS detection, skin samples were embedded in optimal cutting temperature (OCT) compounds and sectioned into 20 μm thick cryo-sections. Sections were incubated with 5 μM MitoSOX at 37 °C for 30 min in the dark. Fluorescence images were captured using a fluorescence microscope (Leica, Deer Park, IL, USA), and fluorescence intensity was quantified using ImageJ software.

For cellular ROS detection, primary KCs were treated with the indicated compounds for 24 h and subsequently incubated with MitoSOX to assess mitochondrial ROS production and oxidative stress levels. Fluorescence intensity was measured using a microplate reader at an excitation wavelength of 488 nm and an emission wavelength of 580 nm.

### 2.9. Histological Examination of H&E Staining

Murine skins were removed, fixed in 10% formaldehyde for three days. Thereafter, the tissues were dehydrated through a series of gradient concentrations of ethanol, cleared with xylene, and embedded in a paraffin medium. Then 5 μm thick sections were prepared by a rotary microtome, and those sections were then stained with hematoxylin and eosin (H&E) for microscopic inspection. The thickness of the epidermis in six random microscopic fields in one slide was measured (in μm) and analyzed using ImageJ software. For each sample, the thickness of the epidermis (from the basal to the granular layer) was measured at 6 different points on section.

### 2.10. Statistical Analysis

Data are presented as the mean ± standard error (SE). Statistical analyses were performed using GraphPad Prism 8.0 software (GraphPad Software, San Diego, CA, USA). One-way or two-way analysis of variance (ANOVA) was applied as appropriate, followed by Bonferroni’s or Newman–Keuls *post hoc* tests for multiple comparisons as appropriate. Mean differences were considered statistically significant when *p* < 0.05.

The severity of skin inflammation was assessed by the Psoriasis Severity Index (PSI) score. Skin erythema, scaling, and thickness were independently regarded as the indicators and each was scored independently on a scale of 0 to 4: 0—none, 1—slight, 2—moderate, 3—marked, 4—very marked. The total PSI score was determined by the cumulative score of all three indicators (Scale 0–12) [20,21].

## 3. Results

### 3.1. The In Vivo Effects of TRPV4 Knockout in IMQ-Induced Psoriasis

#### 3.1.1. Effect of TRPV4 Knockout on IMQ-Induced Akt/mTOR Phosphorylation and ROS Production in Murine Skin Tissue

We first investigated the role of the TRPV4 channel in IMQ-induced psoriasis. Wild-type (WT) and TRPV4 knockout (KO) mice were treated topically on the dorsal skin with either imiquimod (IMQ) or vehicle. After 7 days of treatment (WT + Vehicle, KO + Vehicle, WT + IMQ, and KO + IMQ), mice were euthanized by CO_2_ overdose, and skin tissues were collected for histological and biochemical analyses.

**Figure 1:**
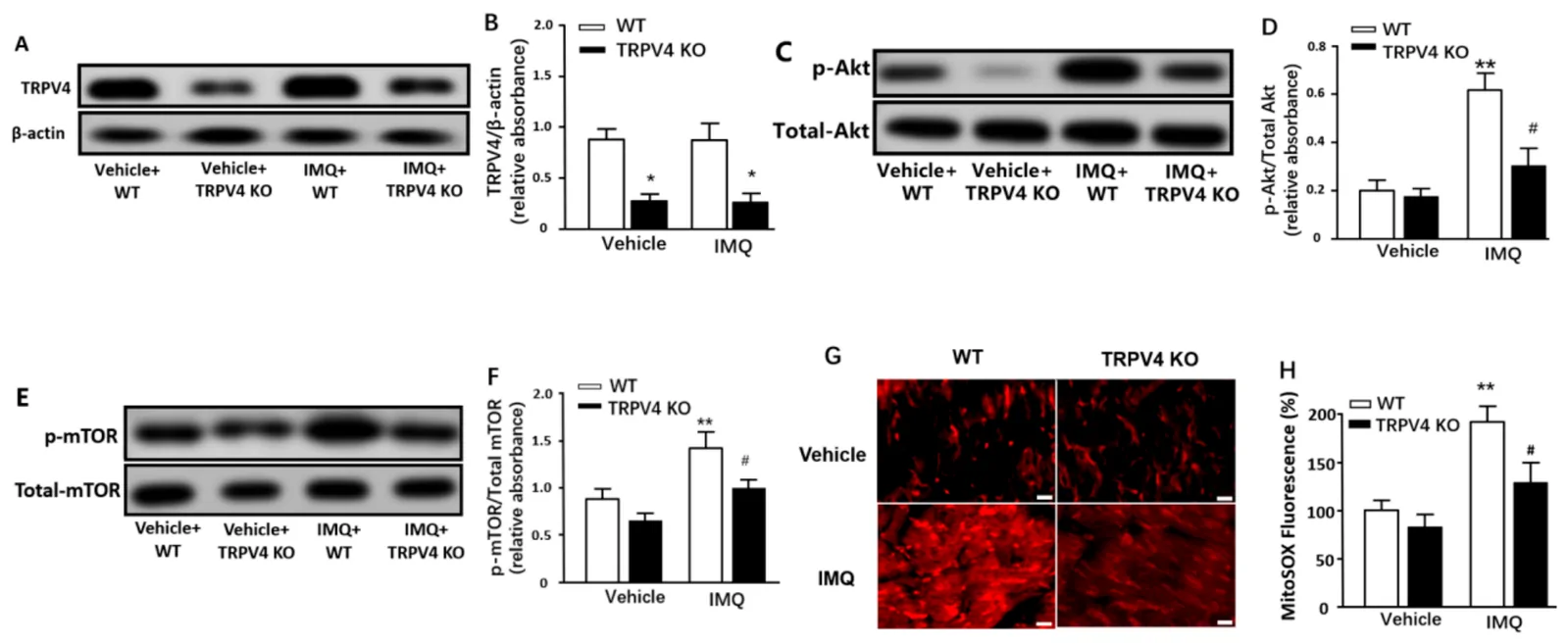
**Effect of TRPV4 knockout on IMQ-induced Akt/mTOR phosphorylation and ROS production in murine skin tissue.** The skin tissues in both wild-type (WT) and TRPV4 KO mice groups that received vehicle or IMQ for 7 days were harvested and analyzed. (**A**) Representative Western blot of skin tissue lysate probed for TRPV4. (**B**) Bar graphs summarizing TRRPV4 protein levels in the murine skin described above. Data are mean ± SE, * *p* < 0.05 as compared with WT mice, n = 3 in each group. (**C**) Representative WWestern blots of skin lysate probed for phosphorylated Akt (p-Akt) and Total Akt. (**D**) Bar graphs summarizing the ratio of p-Akt vs. Total Akt. Data are presented as means ± SE. ** *p* < 0.01 as compared with the group of WT mice group that received vehicle. # *p* < 0.05 as compared with WT mice that received IMQ, n = 3 in each group. (**E**) Represent Western blots of skin lysate probed for phosphorylated mTOR (p-mTOR) and Total-mTOR. (**F**) Bar graphs summarizing the ratio of p-mTOR vs. Total mTOR. Data are presented as means ± SE. ** *p* < 0.01 as compared with the group of WT mice group that received vehicle. # *p* < 0.05 as compared with WT mice that received IMQ, n = 3 in each group. (**G**) Representative microscopic images of ROS production level. Scale bar: 50 μm. (**H**) Bar graphs summarizing the quantitative analysis of ROS production levels measured with the fluorescent probe of MitoSOX, expressed as the percentage of MitoSOX fluorescence. Data are means ± SE. ** *p* < 0.01 vs. WT mice that received vehicle. # *p* < 0.05 vs. WT mice that received IMQ, n = 6 in each group. A two-way ANOVA with Tukey’s multiple comparison test was employed to compare the indexes of all mice.

Western blot analysis was performed to evaluate the effect of TRPV4 gene deletion on TRPV4 expression in murine skin tissues (Figure 1A,B). The results demonstrated that TRPV4 expression was significantly reduced in the skin of TRPV4 KO mice compared with their WT counterparts.

To assess the activity of the calmodulin signaling pathway, the phosphorylation levels of its downstream effectors Akt and mTOR were determined by measuring the ratios of phosphorylated Akt to total Akt (p-Akt/Total Akt) and phosphorylated mTOR to total mTOR (p-mTOR/Total mTOR). As shown in Figure 1C,D, 7 days of IMQ treatment significantly increased the p-Akt/Total Akt ratio in WT mice. In contrast, TRPV4 deletion markedly attenuated IMQ-induced Akt phosphorylation in skin tissues. Similarly, IMQ treatment significantly elevated the p-mTOR/Total mTOR ratio in WT mice (Figure 1E,F), whereas TRPV4 knockout substantially reduced IMQ-induced mTOR phosphorylation.

MitoSOX staining was used to quantify ROS levels (Figure 1G,H). IMQ treatment markedly increased ROS production in WT murine skin, whereas TRPV4 deletion significantly attenuated the IMQ-induced ROS level increment. These findings indicate that genetic ablation of TRPV4 alleviates IMQ-induced oxidative stress in skin tissues.

#### 3.1.2. TRPV4 Deletion Attenuates IMQ-Induced Psoriasis Severity Level and Reduces Inflammatory Cytokine Levels in Mice

We next examined the effects of TRPV4 on the development and severity of IMQ-induced psoriasis. After 7 days of IMQ treatment, mice were euthanized, and dorsal skin were harvested and evaluated.

**Figure 2:**
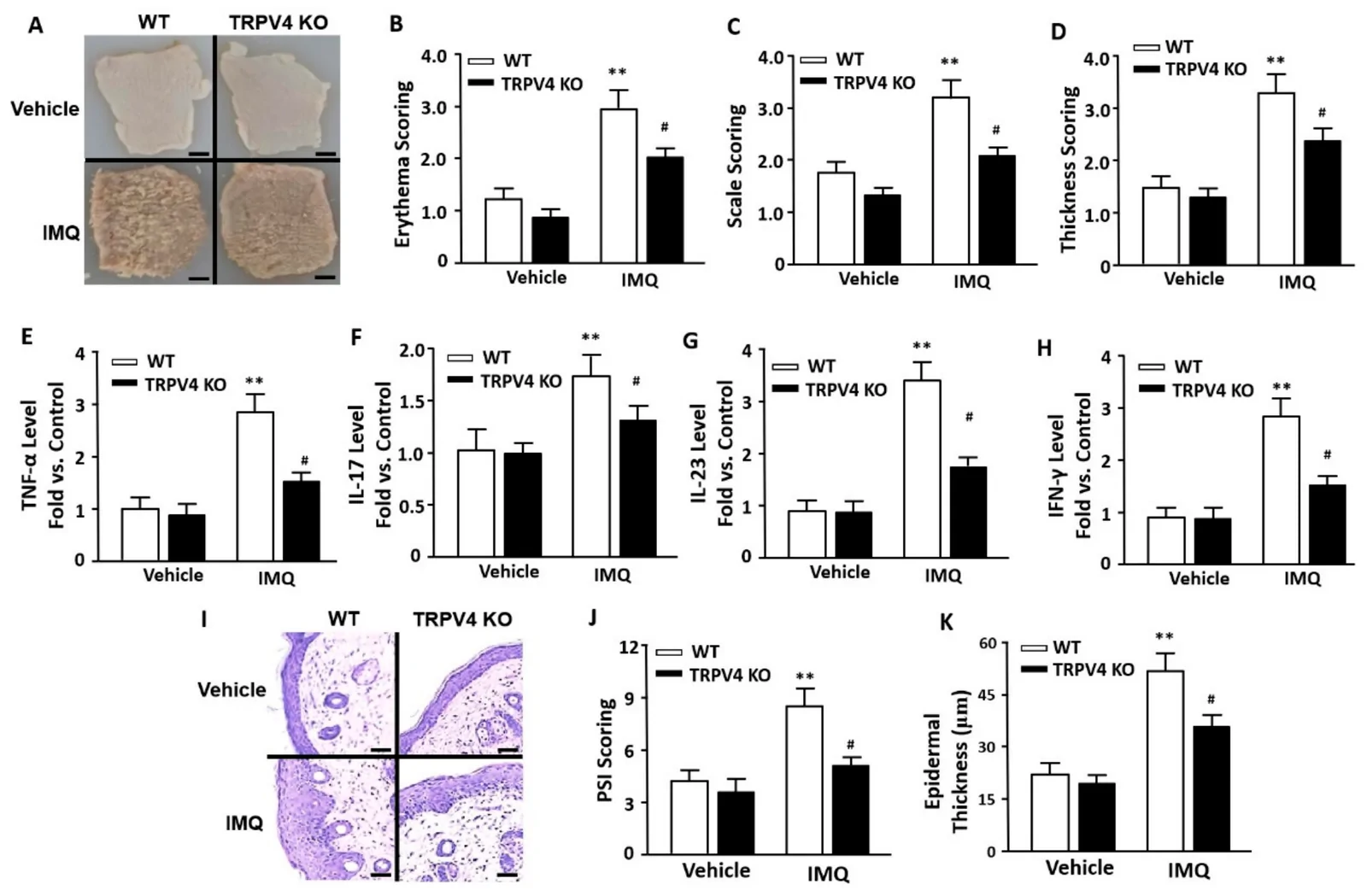
Effect of TRPV4 nullification on IMQ-induced PSI scoring, scale, epidermal thickness and cytokine levels (i.e., TNF-α, IL-17, IL-23 and IFN-γ) in mice. The skin and serum in both WT and TRPV4 KO mice groups that received vehicle or IMQ for 7 days were collected, scored and analyzed. (**A**) Representative dermal morphological alterations from WT or TRPV4 KO mice that received vehicle or IMQ for 7 days under such conditions within four groups. Scale bar: 2 mm. (**B**) Bar graphs summarizing the erythema scoring (0–4 points) of murine skin within four groups after 7-day IMQ treatment. Data are presented as means ± SE, ** *p* < 0.01 as compared with the group of vehicle in WT mice. # *p* < 0.05 as compared with the group of IMQ in WT mice, n = 6 in each group. (**C**) Bar graphs summarizing the scale scoring (0–4 points) of murine skin within four groups after 7-day IMQ treatment. Data are mean ± SE, ** *p* < 0.01 as compared with the group of Vehicle + WT. # *p* < 0.05 as compared with the group of IMQ + WT, n = 6 in each group. (**D**) Bar graphs summarizing the thickness scoring (0–4 points) of murine skin within four groups after 7-day IMQ treatment. Data are mean ± SE, **p < 0.01 as compared with the group of Vehicle + WT. # *p* < 0.05 as compared with the group of IMQ + WT, n = 6 in each group. (**E**) Bar graphs summarizing the TNF-α level in murine serum within four groups. Data are mean ± SE, ** *p* < 0.01 as compared with the group of Vehicle + WT. # *p* < 0.05 as compared with the group of IMQ + WT, n = 6 in each group. (**F**) Bar graphs summarizing the murine serum IL-17 level within four groups. Data are mean ± SE, ** *p* < 0.01 as compared with the group of Vehicle + WT. # *p* < 0.05 as compared with the group of IMQ + WT, n = 6 in each group. (**G**) Bar graphs summarizing the murine serum IL-23 level within four groups. Data are mean ± SE, **p < 0.01 as compared with the group of Vehicle + WT. # *p* < 0.05 as compared with the group of IMQ + WT, n = 6 in each group. (**H**) Bar graphs summarizing the murine serum IFN-γ level within four groups. Data are mean ± SE, ** *p* < 0.01 as compared with the group of Vehicle + WT. # *p* < 0.05 as compared with the group of IMQ + WT, n = 6 in each group. (**I**) Representative microscopic images showing histological alterations from WT or TRPV4 KO mice that received vehicle or IMQ for 7 days within four groups. Scale bar: 25 μm. The average of cytokine expression in Vehicle + WT group is normalized as 1-fold. (**J**) Bar graphs summarizing the PSI scoring of murine skin within four groups after 7-day IMQ treatment as the cumulative value of erythema, scale and thickness scoring. Data are presented as means ± SE, ** *p* < 0.01 as compared with the group of vehicle in WT mice. # *p* < 0.05 as compared with the group of IMQ in WT mice, n = 6 in each group. (**K**) Bar graphs summarizing the epidermal thickness (μm) of murine skin within four groups after 7-day IMQ treatment. Data are mean ± SE, ** *p* < 0.01 as compared with the group of Vehicle + WT. # *p* < 0.05 as compared with the group of IMQ + WT, n = 6 in each group. A two-way ANOVA with Tukey’s multiple comparison test was employed to compare the indexes of all mice.

No signs of dermatitis or significant changes in psoriasis-related indexes were observed in either WT or KO mice treated with vehicle alone. In contrast, WT mice treated with IMQ developed severe psoriasiform symptoms, including erythema, adherent scaling and epidermal thickening (Figure 2A–D,I–K). Notably, these pathological manifestations were substantially alleviated in TRPV4 KO mice following IMQ treatment. These findings suggest that suppression of TRPV4 expression attenuates the severity of IMQ-induced psoriasiform dermatitis.

To further evaluate inflammatory responses, serum levels of four representative psoriasis-associated cytokines, including TNF-α, IL-17, IL-23, and IFN-γ, were measured. IMQ treatment markedly increased the levels of all four cytokines in WT mice. However, these cytokine levels were significantly reduced in TRPV4 KO + IMQ mice compared with the WT + IMQ mice group (Figure 2E–H). Collectively, these results suggest that TRPV4 inhibition might regulate the development and progression of IMQ-induced psoriasis.

### 3.2. The In Vitro Effects of Downstream TRPV4 Signaling Pathways in IMQ-Treated KCs

#### 3.2.1. Effects of IMQ Concentration on Autophagy, Cell Proliferation, and ROS Production in Primary KCs

Accumulating evidence indicates that autophagy plays an important role in the pathophysiological progression in IMQ-induced psoriasis. As a critical intracellular protective mechanism, autophagy removes damaged organelles, such as mitochondria, and ROS-generating proteins, thereby preventing the accumulation of harmful cellular components that contribute to psoriasis.

**Figure 3:**
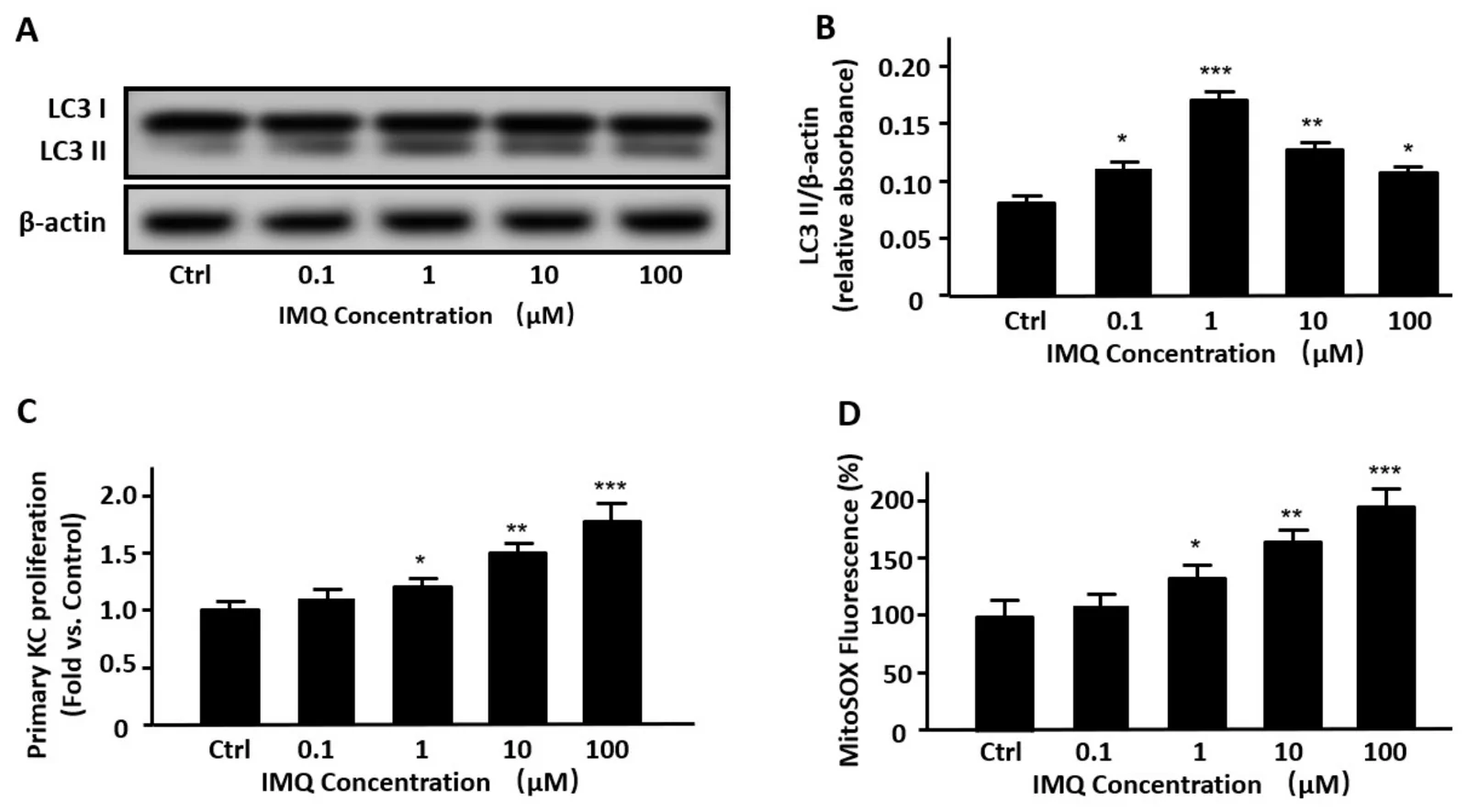
**Effect of IMQ with different concentrations on autophagy, cellular proliferation and ROS production in primary KCs.** The cells were exposed under different IMQ concentrations for 24 h, then autophagy, proliferation and ROS level were measured. (**A**) Representative Western blot of cultured KC applied with anti-LC3 antibodies after treatments with HBSS control, and IMQ at different concentrations (ranging from 0.1 to 100 μM) to measure the extent of autophagy. (**B**) Bar graphs showing the effect on autophagy under IMQ exposure with different concentrations. Data are mean ± SE. * *p* < 0.05 vs. Ctrl, ** *p* < 0.01 vs. Ctrl, *** *p* < 0.001 vs. Ctrl, n = 3 in each group. (**C**) Bar graphs showing the effect on primary KC proliferation level indicating the mitochondria ROS production of primary KC. * *p* < 0.05 vs. Ctrl, ** *p* < 0.01 vs. Ctrl, *** *p* < 0.001 vs. Ctrl, n = 6 in each group. (**D**) Bar graphs showing the effect on MitoSOX fluorescence level indicating the mitochondria ROS production of primary KCs. Data are mean ± SE, * *p* < 0.05 vs. Ctrl, ** *p* < 0.01 vs. Ctrl, *** *p* < 0.001 vs. Ctrl, n = 6 in each group. A one-way ANOVA with Tukey’s multiple comparison test was employed to compare the indexes of all mice.

To investigate the effects of IMQ on autophagy in KCs, autophagic activity was assessed by Western blot analysis. The results showed that IMQ induced a biphasic effect on autophagy. Specifically, autophagy was enhanced at low IMQ concentrations (0.1~1 μM) but declined at higher concentrations (1~100 μM) (Figure 3A,B). In addition, CCK-8 assays demonstrated that IMQ promoted KC proliferation in a dose-dependent manner (Figure 3C). Consistently, MitoSOX fluorescence staining revealed a dose-dependent increase in intracellular ROS levels following IMQ treatment (Figure 3D).

#### 3.2.2. Effects of the Calmodulin/Akt Pathway on IMQ-Induced Autophagic Alterations, ROS Production, and Proliferation of KCs

Increasing evidence suggests that mitochondrial ROS production plays a critical role in the progression of IMQ-induced psoriasis. To determine whether the Calmodulin/Akt pathway is involved in IMQ-induced autophagic regulation, cultured KCs were treated with IMQ (100 μM) or vehicle in the presence or absence of AZD5363 (1 μM), the Akt inhibitor or W-7 (alias as HY-100912, 1 μM), the calmodulin inhibitor, for 24 h.

**Figure 4:**
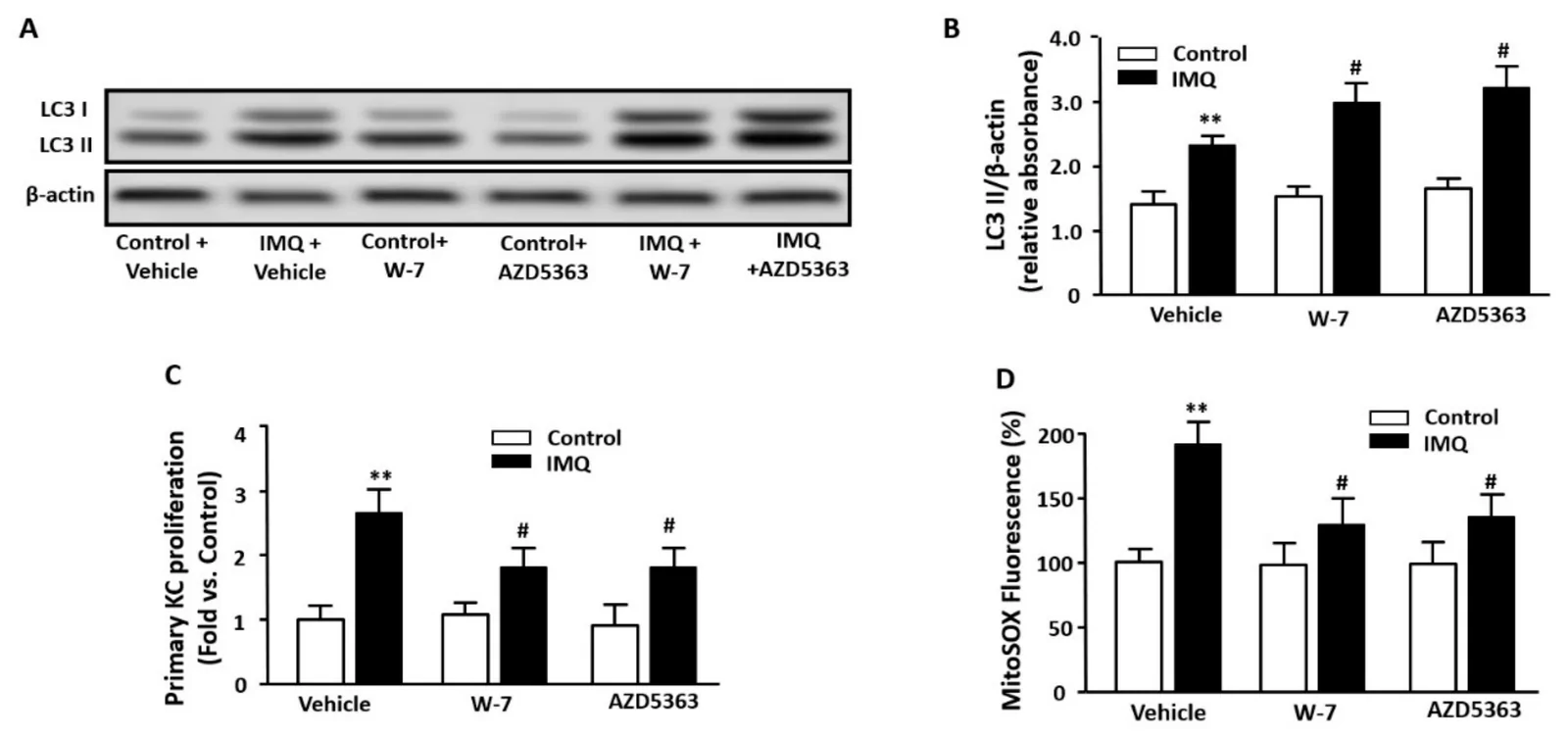
Effect of IMQ and blockade of Calmodulin/Akt signaling pathway on autophagy, cell proliferation and ROS production in primary KCs. Autophagy, cell proliferation and ROS production in primary KCs were examined with vehicle control, IMQ (100 μM) with or without the Calmodulin inhibitor, W-7 (1 μM) or the Akt inhibitor, AZD5363 (1 μM) for 24 h. (**A**) Representative Western blot results of cultured primary KCs treated with MAP-LC3 antibody for autophagy after relevant treatments. (**B**) Bar graphs summarizing quantitative analysis of autophagic alterations in primary KCs treated under the conditions as described above. Data are mean ± SE, ** *p* < 0.01 as compared with the group of Vehicle + Control, # *p* < 0.05 as compared with the group of Vehicle + IMQ, n = 3 in each group. (**C**) Bar graphs showing the effect on primary KC proliferation level indicating the extent of psoriasis with the conditions described above. Data are mean ± SE, ** *p* < 0.01 as compared with the group of Vehicle + Control, # *p* < 0.05 as compared with the group of Vehicle + IMQ, n = 10 in each group. (**D**) Representative fluorescence micrographs of cultured primary KC probed by MitoSOX after treatments for mitochondria ROS. Data are mean ± SE, ** *p* < 0.01 as compared with the group of Vehicle + Control. # *p* < 0.05 as compared with the group of Vehicle + IMQ, n = 10 in each group. A two-way ANOVA with Tukey’s multiple comparison test was employed to compare the indexes of all mice.

As shown in Figure 4A,B, IMQ treatment significantly enhanced autophagy in KCs. Pretreatment with either AZD5363 or W-7 further increased IMQ-induced autophagic activity. In contrast, treatment with AZD5363 or W-7 alone did not affect basal autophagy levels. These findings indicate that inhibition of the Calmodulin/Akt pathway enhances IMQ-induced autophagy, suggesting that this signaling pathway negatively regulates autophagy in KCs.

To further determine whether activation of the Calmodulin/Akt pathway contributes to psoriasis progression, KC proliferation and ROS production were evaluated following exposure to IMQ (100 μM) or vehicle. As shown in Figure 4C,D, IMQ significantly increased both cell proliferation and ROS production. Pretreatment with AZD5363 or W-7 markedly attenuated those IMQ-induced effects. Neither inhibitor alone affected basal proliferation or ROS levels in KCs. These results suggest that inhibition of the Calmodulin/Akt pathway suppresses IMQ-induced proliferation and oxidative stress in KCs, indicating that activation of this pathway contributes to psoriasis progression.

#### 3.2.3. Role of the CaMKKβ/AMPK Pathway in IMQ-Induced Autophagic Alterations, ROS Production, and Proliferation of KCs

Previous studies suggested that the CaMKKβ/AMPK signaling pathway, one downstream effector of TRPV4, might also participate in the development of IMQ-induced psoriasis. We therefore investigated whether the protective effects of CaMKKβ/AMPK pathway against IMQ-induced ROS production are mediated through the promotion of autophagy and subsequent clearance of damaged mitochondria, one of the major intracellular sources of ROS.

**Figure 5:**
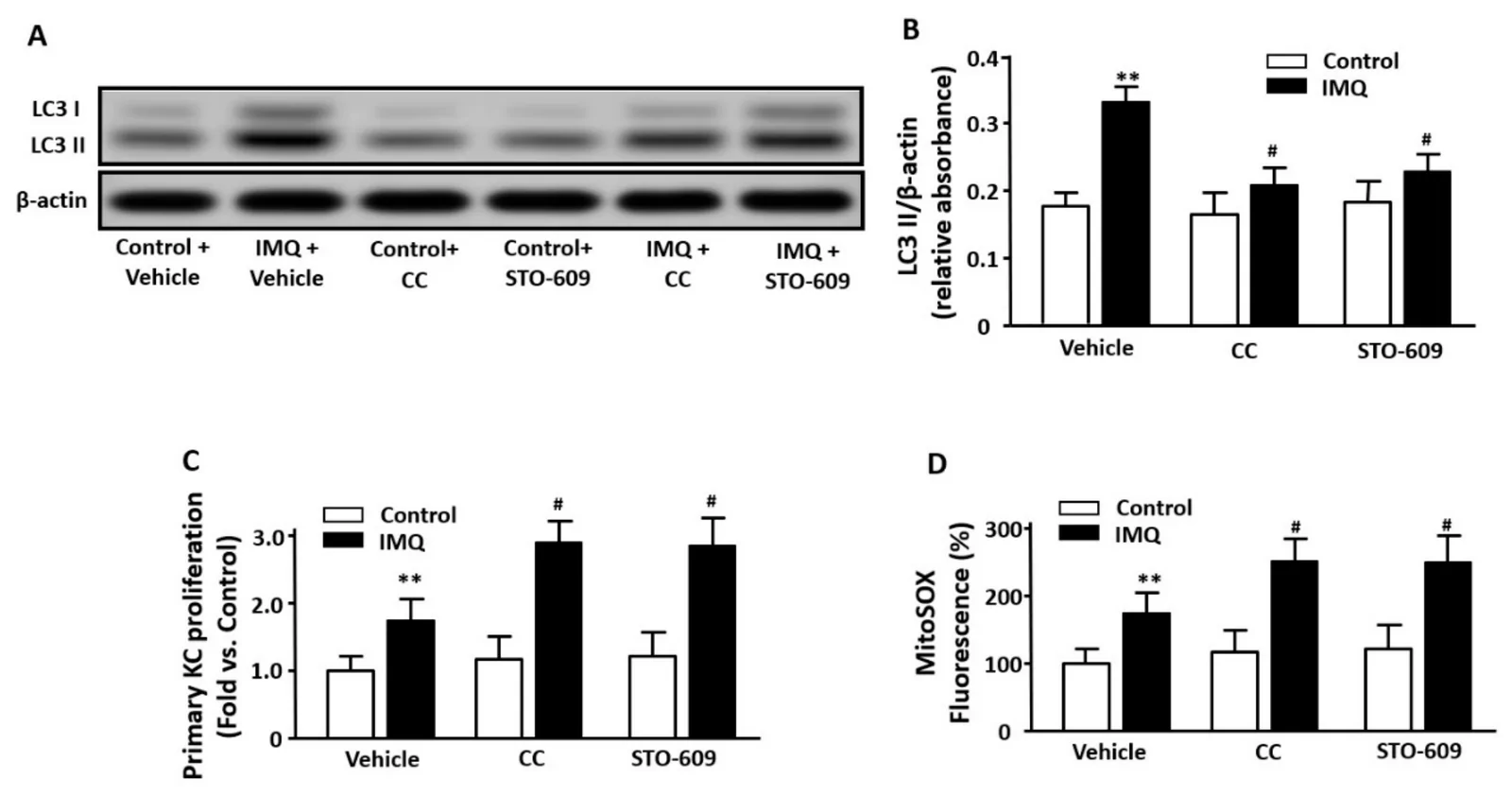
Effect of IMQ and blockade of CaMKKβ/AMPK signaling pathway on autophagy, cell proliferation and ROS production in primary KC. Autophagy, cell proliferation and ROS production in primary KCs were examined with vehicle control, IMQ (100 μM) with or without the AMPK inhibitor of Compound C (CC, 1 μM) or the CaMKK inhibitor of STO-609 (1 μM) pretreatment for 24 h. (**A**) Representative Western blot results of cultured primary KCs applied with MAP-LC3 antibody after relevant treatments. (**B**) Bar graphs summarizing quantitative analysis of autophagic alterations in primary KCs treated under the conditions described above. Data are mean ± SE, ** *p* < 0.01 as compared with the group of Vehicle + Control. # *p* < 0.05 as compared with the group of Vehicle + IMQ, n = 3 in each group. (**C**) Bar graphs showing the effect on primary KC proliferation level indicating the extent of psoriasis with the conditions described above. Data are mean ± SE, ** *p* < 0.01 as compared with the group of Vehicle + Control. # *p* < 0.05 as compared with the group of Vehicle + IMQ, n = 10 in each group. (**D**) Representative fluorescence micrographs of cultured primary KC probed by MitoSOX after treatments for mitochondria ROS. Data are mean ± SE, ** *p* < 0.01 as compared with the group of Vehicle + Control. # *p* < 0.05 as compared with the group of Vehicle + IMQ, n = 10 in each group. A two-way ANOVA with Tukey’s multiple comparison test was employed to compare the indexes of all mice.

To determine the role of the CaMKKβ/AMPK pathway in IMQ-induced autophagy, KCs were pretreated with STO-609 (1 μM, the CaMKKβ inhibitor) or Compound C (CC, 1 μM, the AMPK inhibitor) before exposure to IMQ (100 μM) or vehicle. As shown in Figure 5A,B, IMQ treatment significantly increased autophagic activity in KCs. However, pretreatment with either STO-609 or CC markedly suppressed IMQ-induced autophagy. Treatment with either inhibitor alone had no significant effect on basal autophagic activity. These findings indicate that inhibition of the CaMKKβ/AMPK pathway attenuates IMQ-induced autophagy, suggesting that this pathway might positively regulate autophagic activity in KCs.

To further evaluate the functional significance of this pathway, KC proliferation and ROS production were assessed following the treatment with IMQ in the presence or absence of STO-609 or CC. As shown in Figure 5C,D, IMQ significantly increased both cell proliferation and ROS production. Pretreatment with STO-609 or CC further exacerbated those IMQ-induced effects. Neither inhibitor alone altered basal proliferation or ROS levels. These results demonstrate that inhibition of the CaMKKβ/AMPK pathway enhances IMQ-induced KC proliferation and oxidative stress, suggesting that activation of this pathway suppresses the progression of IMQ-induced psoriasis.

## 4. Discussion

The present study investigated the role of TRPV4 in psoriasis and provided potential evidence that two downstream signaling pathways of TRPV4, namely the CaMKKβ/AMPK and Calmodulin/Akt pathways, participate in the initiation and progression of IMQ-induced psoriasis through autophagic and oxidative stress regulation. Our findings demonstrated that activation of the Calmodulin/Akt pathway suppresses autophagic activity and enhances mitochondrial ROS production, whereas triggering the CaMKKβ/AMPK pathway exerts a protective effect by promoting autophagy-mediated clearance of mitochondrial ROS, thereby decelerating the progression of IMQ-induced psoriasis in mice.

TRP channel family members mediate calcium influx into cells, and several TRP channels are functionally expressed in KCs. These channels are involved in both physiological and pathological processes of skin diseases, including psoriasis [7,9]. Hyperforin-activated TRPC6 is responsible for differentiation of KCs, which is akin to solely Ca^2+^ stimulation [22]. TRPV4, which regulates KC proliferation and differentiation, activates the downstream signaling pathways such as Akt, MAPK and extracellular signal-regulated kinase (ERK) through calcium influx-mediated mechanisms [23,24]. In the present study, TRPV4 knockout markedly reduced the severity of psoriasiform dermatitis, epidermal thickness, and the number of proliferating KCs in IMQ-treated mice. These findings suggest that TRPV4 contributes to the pathogenesis of psoriasis by promoting proliferation of KCs. Recent studies have revealed constitutive activation of STAT3 in both human psoriatic lesions and transgenic murine KCs, and persistent STAT3 activation in KCs is sufficient to induce psoriasiform phenotypes [25]. Given the important role of TRPV4 in KC proliferation and differentiation, it is likely to be involved in psoriasis development. Moreover, emerging evidence indicates that TRP channels expressed in sensory C-fibers contribute to neurogenic inflammation through the release of pro-inflammatory neuropeptides, including substance P (SP) and calcitonin gene-related peptide (CGRP) [26,27]. SP mediates itch sensation and stimulates IL-1 and TNF-α synthesis, whereas CGRP possesses potent vasodilatory activity and is required for the polarization of immune responses toward the Th17 phenotype. TRPV4 activation stimulates SP and CGRP release in murine bladder and airway tissues [28]. In addition, inhibition of neuropeptide release by botulinum toxin significantly alleviates psoriasiform dermatitis in IMQ-treated mice [21,29]. IMQ, primarily known as a potent agonist of Toll-like receptors 7 and 8 (TLR7/8), extensively participates in the crosstalk between TLR pathways and TRP channels with inflammatory cascades, characterized by the rapid release of pro-inflammatory mediators such as TNF-α, IL-1, and the IL-23/IL-17 axis instead of a solely off-target effect. These inflammatory cytokines, along with associated oxidative stress in the epidermal microenvironment, are well-documented sensitizers and transcriptional stimulators of TRPV4 in various cell types, including keratinocytes and immune cells [14,30]. Collectively, these observations suggest that TRPV4 might promote psoriasis progression through modulation of neurogenic inflammation, including neural fiber activation and enhanced neuropeptide secretion.

Oxidative stress results from the imbalance between ROS production and antioxidant defense systems. Extensive evidence proved that dysfunction of antioxidant systems coupled with excessive ROS production contributes to the pathogenesis of numerous diseases [31]. The antioxidant defense system consists of several enzymatic complexes, including catalase (CAT), superoxide dismutase (SOD), and glutathione peroxidase (GSH-Px) [32]. Elevated ROS production and impaired antioxidant activity are involved in psoriasis development. Indeed, studies over the past decade have shown increased levels of ROS, including nitric oxide (NO^•^), hydrogen peroxide (H_2_O_2_), and superoxide anion (O_2_^•−^), in psoriatic skin lesions [33]. Consequently, targeting oxidative stress has emerged as a promising therapeutic strategy for psoriasis. Antioxidants such as curcumin, ascorbic acid, α-tocopherol, and coenzyme Q10 could enhance endogenous antioxidant defenses, counteract ROS accumulation, prolong cellular lifespan, and delay aging processes, thereby contributing to psoriasis prevention and treatment. SIRT1 is also proposed as a therapeutic target for psoriasis, since restoration of SIRT1 activity improves mitochondrial function and redox homeostasis through modulation of MAPK signaling, ultimately reducing ROS production [34]. Moreover, probiotics have been reported to possess antioxidant properties, and appropriate probiotic supplementation might improve disease progression by alleviating oxidative stress [35]. Taken together, these findings indicate that current pharmacological therapies, healthy lifestyles, and antioxidant-rich diets may collectively reduce oxidative damage and slow psoriasis progression, and the present study further supports this concept by demonstrating that IMQ significantly increases mitochondrial ROS production in KCs.

Elevated mitochondrial ROS levels were closely associated with impaired autophagic function. Autophagy serves as a critical intracellular protective mechanism responsible for the elimination of damaged organelles and ROS-generating cellular components. Autophagy eliminates misfolded proteins and dysfunctional organelles, particularly damaged mitochondria, and maintains cellular homeostasis [36]. Furthermore, the p62–KEAP1–NRF2 signaling axis, a highly conserved cellular defense mechanism, couples selective autophagy with the antioxidant stress response, delicately regulating the damaged protein clearance to combat ROS within the transcriptional defense [37]. The intracellular transcription factor NRF2 is relatively low under normal status, while oxidative stress makes it heterodimerize with tiny Maf proteins, binding to the target genes and compelling a series of cytoprotective genes such as NQO1 and HO-1 to function, including targeted degradation with a positive feedback [38]. Accumulation of dysfunctional mitochondria increases intracellular ROS levels, triggers abnormal protein synthesis and genetic modification, ultimately contributing to the development of psoriasis and other immune-associated disorders.

Several downstream signaling pathways have been identified for TRPV4. First, TRPV4 functions as a Ca^2+^ influx channel, leading to increased intracellular Ca^2+^ concentrations that directly regulate autophagy initiation and flux. Second, the CaMKKβ–AMPK–mTOR pathway represents a classical autophagy-promoting mechanism, in which CaMKKβ activates AMPK and subsequently inhibits mTOR to induce autophagy. Third, the Ca^2+^–Calcineurin–TFEB axis enhances autophagy and lysosomal biogenesis through Calcineurin-mediated dephosphorylation of TFEB, leading to its nuclear translocation. Finally, excessive TRPV4 activation might disrupt Ca^2+^ homeostasis, resulting in endoplasmic reticulum stress, mitophagy dysfunction, and mitochondrial injury [10,39]. Ca^2+^ allosterically activates the upstream kinase CaMKKβ, leading to AMPK phosphorylation and activation, independent of AMP/ATP ratio alterations. Specifically, CaMKKβ directly phosphorylates the Thr172 residue of AMPK, thereby activating AMPK and promoting autophagy through multiple mechanisms [40]. Conversely, excessive TRPV4 activation may impair autophagic function, cause Ca^2+^ dysregulation and subsequent imbalance of the AMPK/mTOR signaling axis, ultimately exacerbating inflammatory responses. In contrast to the CaMKKβ/AMPK pathway, the Calmodulin/Akt pathway generally functions as a negative regulator of autophagy. This observation highlights the bidirectional nature of TRPV4-mediated calcium signaling in autophagy regulation. Calcium signaling activates Akt (protein kinase B, PKB) through phosphorylation of the Thr308 residue [41,42]. It is commonly acknowledged for long that the PI_3_K/Akt/mTOR axis facilitates the inhibition of pro-psoriatic effect and Akt is a serine/threonine kinase belonging to the AGC kinase family (including PKA, PKC, and PKG) and regulates numerous cellular processes, including cell survival, apoptosis inhibition, and glucose metabolism through phosphorylation of multiple downstream substrates [43]. Akt activity is tightly controlled by phosphorylation at both the activation loop and C-terminal regulatory domains [44]. Activation of Akt is typically associated with growth and survival signaling initiated by receptor–ligand interactions on cell surface [45]. Moreover, Calmodulin can indirectly enhance Akt activation through interactions with PI_3_K and promote mTORC1activation, thereby suppressing autophagy [42,46]. In the present study, inhibition of the CaMKKβ/AMPK pathway significantly attenuated the autophagic response induced by IMQ stimulation, resulting in elevated mitochondrial ROS production and enhanced KC proliferation. These findings indicate that the CaMKKβ/AMPK pathway exerts protective effects in IMQ-induced psoriasis by promoting autophagy and suppressing mitochondrial ROS accumulation. The only application of LC3 is a limitation, as it would be more favorable to consolidate our conclusions by performing experiments regarding autophagic influx to present dynamic views of the formation of autophagosome and its fusion with lysosomes.

Finally, inhibition of TRPV4 significantly reduced the severity of dermatitis, epidermal hyperplasia, and inflammatory cell infiltration in IMQ-induced psoriasiform lesions [14]. These findings support the notion that TRPV4 antagonists may serve as promising therapeutic agents in animals by modulating epidermal proliferation, inflammatory responses, and oxidative stress. Taken together, our results suggest that TRPV4 contributes to the pathogenesis of psoriasis by regulating proliferation and ROS production in KCs through activation of the CaMKKβ/AMPK and Calmodulin/Akt signaling pathways in a dose-dependent manner.

## 5. Conclusions

**Figure 6:**
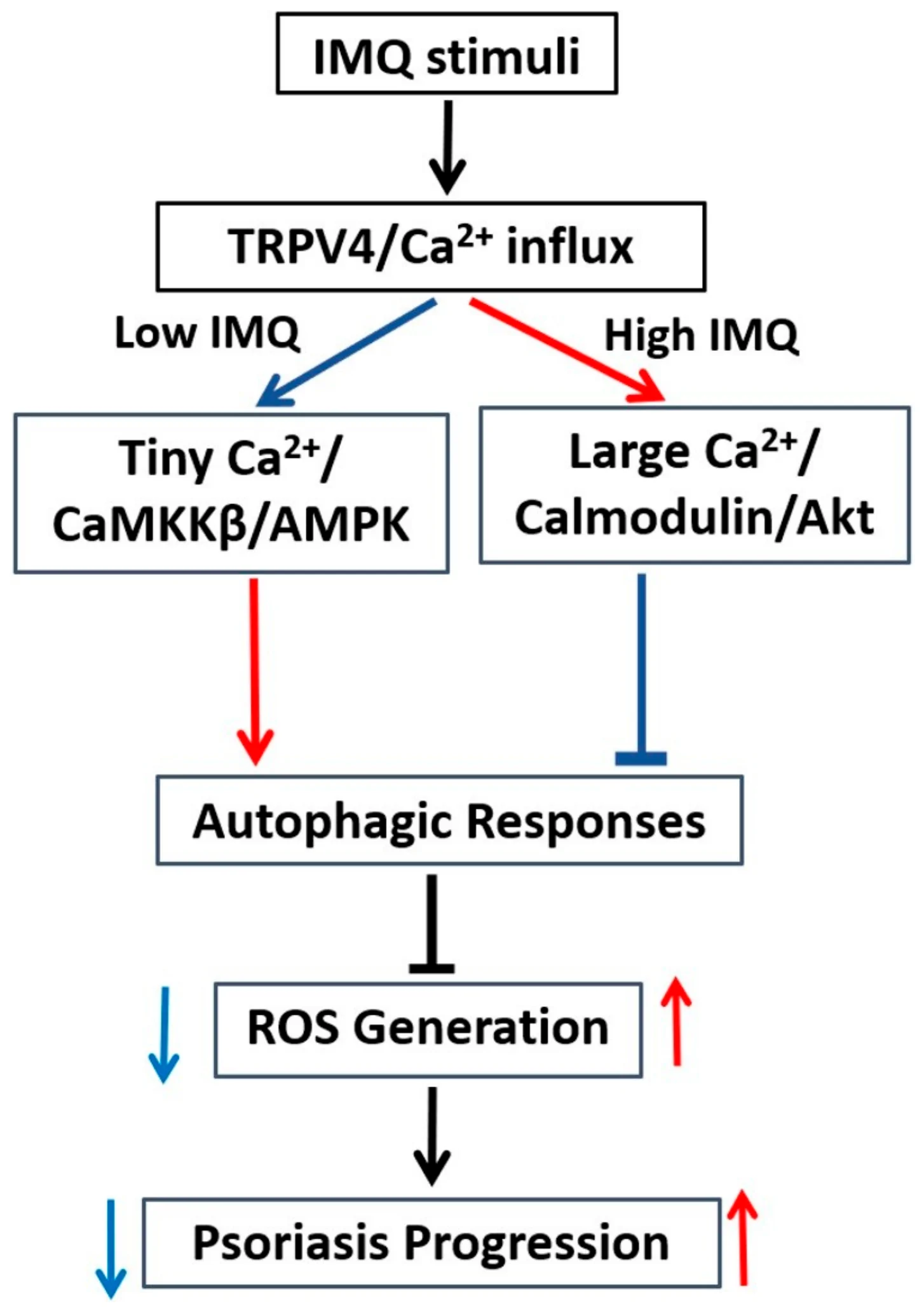
Proposed intracellular mechanisms involved in the activation of CaMKKβ/AMPK vs. Calmodulin/Akt signaling pathway of IMQ-induced psoriasis in KCs.

TRPV4-associated signaling pathways participate in the pathogenesis of IMQ-induced psoriasis, with both the CaMKKβ/AMPK and Calmodulin/Akt pathways playing essential roles. At relatively lower IMQ concentrations, the CaMKKβ/AMPK pathway is preferentially activated, leading to enhanced autophagy as a compensatory response that partially counteracts psoriasis progression. As IMQ concentration continuously increases, activation of the Calmodulin/Akt pathway becomes dominant, resulting in ROS accumulation, accelerated psoriasis progression, and enhanced immunological dysregulation (Figure 6).

This conclusion is supported by several pieces of evidence: (1) Inhibition of Calmodulin/Akt pathway enhanced IMQ-induced autophagy, whereas suppression of CaMKKβ/AMPK pathway attenuated such autophagic response. (2) Blockade of Calmodulin/Akt pathway reduced IMQ-induced mitochondrial ROS accumulation and alleviated psoriasis development. (3) Inhibition of the CaMKKβ/AMPK pathway exacerbated mitochondrial ROS production and aggravated psoriasis progression. (4) TRPV4 knockout significantly attenuated IMQ-induced psoriasiform lesions, reduced psoriasis-associated cytokine secretion, suppressed Akt/mTOR phosphorylation, and decreased ROS production in skin tissues.

## Data Availability

The raw data supporting the conclusions of this article will be made available by the authors on request.
